# Some Key Factors Affecting Consumers’ Intentions to Purchase Functional Foods: A Case Study of Functional Yogurts in Vietnam

**DOI:** 10.3390/foods9010024

**Published:** 2019-12-25

**Authors:** Ninh Nguyen, Hoang Viet Nguyen, Phuong Thao Nguyen, Viet Thao Tran, Hoang Nam Nguyen, Thi My Nguyet Nguyen, Tuan Khanh Cao, Tran Hung Nguyen

**Affiliations:** 1 Department of Entrepreneurship, Innovation and Marketing, La Trobe Business School, La Trobe University, Bundoora VIC 3086, Australia; 2 Business Sustainability Research Group, Thuongmai University, 79 Ho Tung Mau Road, Hanoi 100000, Vietnam; 3 Department of Research Administration, Thuongmai University, 79 Ho Tung Mau Road, Hanoi 100000, Vietnam; tranvietthao@tmu.edu.vn; 4 Department of Social Sciences, Luong The Vinh School, Hanoi 100000, Vietnam; 5 Department of Strategic Management, Thuongmai University, 79 Ho Tung Mau Road, Hanoi 100000, Vietnam; namqtcl@tmu.edu.vn (H.N.N.); mynguyet@tmu.edu.vn (T.M.N.N.); 6 Department of Marketing Management, Thuongmai University, 79 Ho Tung Mau Road, Hanoi 100000, Vietnam; khanhct@tmu.edu.vn; 7 Faculty of Economic Information System and Electronic Commerce, Thuongmai University, 79 Ho Tung Mau Road, Hanoi 100000, Vietnam; hung.tmdt@tmu.edu.vn

**Keywords:** functional foods, functional yogurts, intention, attitude, subjective norm, health consciousness, price, Vietnam

## Abstract

The development of functional foods is key to promoting a healthy diet and preventing certain diseases. This study aims to examine several key factors that affect consumer attitude and intention with respect to purchasing functional foods in an emerging market economy. A research model was developed by extension of the Theory of Reasoned Action (TRA), and then validated through obtaining data from 596 Vietnamese consumers who were interested in functional yogurts using an interviewer-administered questionnaire. Multivariate data analysis reveals that while health consciousness and subjective norm significantly enhance consumers’ attitudes towards purchasing functional yogurts, perceived price of functional yogurts exerts a negative impact on such attitudes. Moreover, subjective norm and attitude appear to be key predictors of consumers’ intentions to buy functional yogurts. These findings extend the extant literature relating to functional food purchase and consumption in emerging markets, and they have several important practical implications for functional yogurt manufacturers, retailers, and policymakers. A major implication is that education and communication programs that aim at increasing consumers’ health consciousness and their awareness of functional foods’ health benefits play an integral role in the success of functional food products such as functional yogurts. Research limitations and future research directions are also presented.

## 1. Introduction

The development of functional foods plays an essential role in the promotion of healthy diet and prevention of certain diseases [1,2]. Functional foods include “food products which have been modified to include a health benefit beyond the traditional nutrients it would normally contains” [1] (p. 6). According to the International Life Sciences Institute, “a food can be regarded as functional if it is satisfactorily demonstrated to affect beneficially one or more target functions in the body, beyond adequate nutritional effects, in a way that is relevant to either an improved state of health and well-being and/or reduction of risk of disease” [3] (p. 5). Functional foods are believed to benefit consumers’ well-being and improve their quality of life [4,5].

Functional food products have become progressively popular in many countries around the world [6]. The size of the global market for functional foods accounted for USD 161.49 billion in 2018, and it is projected to reach a value of USD 275.77 billion by 2025 [7]. Understanding how and why consumers buy functional foods is of great importance for the sustainable development of the functional food sector [8]. Therefore, a considerable number of research studies have sought to examine and explain consumer purchase intention, acceptance, and consumption with reference to functional foods [9,10]. Such studies have highlighted relevant influencing factors including demographics factors (e.g., age and gender), psychological factors (e.g., knowledge, perceived benefits, concerns about health, safety and nutrition, and attitudes), situational factors (e.g., price and availability) and socio-cultural factors [11,12,13,14,15]. Nevertheless, the majority of past research focuses on developed and Western countries, especially those in Europe and North America. Hence, their findings may not be applicable to other socioeconomic contexts such as developing and emerging market economies [16]. For example, while Huang et al. [16] assert that the high price of functional foods reduces intention to purchase these products among Chinese consumers, Pappalardo and Lusk [14] find that Italian consumers are willing to pay more for a functional snack if the product’s health benefits satisfy their requirement. Therefore, more research is needed to provide new insights into consumer behavior towards functional foods in emerging markets, particularly those in Asia, like Vietnam.

Vietnam with a population of 96.2 million [17] and rising consumer concerns about health and food-related issues [18,19] represents a colossal market opportunity for healthy food products such as functional foods. In fact, functional food is currently the largest health and wellness product category in the country [20]. The latest data from Euromonitor International [20] shows that the sales of functional packaged foods have continuously increased from VND 43.2 trillion in 2013 to VND 69.9 trillion in 2018. Vietnamese consumers are particularly interested in functional dairy products, baby food, cereals, biscuits, and vegetables. The functional food market has attracted both domestic and international players with the market leader being Vietnam Dairy Products (Vinamilk, Ho Chi Minh City, Vietnam). The local firm dominates the sector with a market share of 41.6%. While functional food firms have been trying to increase consumer demand, little knowledge is known about consumer attitude, intention, and behavior towards functional foods in Vietnam.

This study, therefore, aims to advance the literature by investigating some key factors affecting consumer attitude and intention with respect to the purchase of functional foods, with a specific focus on functional yogurts, in the emerging market context of Vietnam. To this end, we will develop and validate a unique research model using data obtained from Vietnamese consumers who are interested in functional yogurts. To the best of our knowledge, our study is among the first attempt to explore and explain how Vietnamese consumers form their attitudes and intentions towards buying functional foods including functional yogurts. Hence, the fresh insights provided by our study will expectedly assist functional food manufacturers and retailers as well as policy makers in the development of effective strategies and campaigns to promote the development of functional foods and the purchase of these products.

The remainder of our paper is organized as follows. In the succeeding section, we discuss the theoretical framework for our study and develop relevant hypotheses. Thereafter, we present the research methods including the focused product category, survey, sample, and analytical method. Then, we provide a discussion of the study findings and their implications. Lastly, we present key concluding remarks and possible directions for future studies.

## 2. Theoretical Framework and Hypotheses

### 2.1. Theoretical Framework

**Figure 1:**
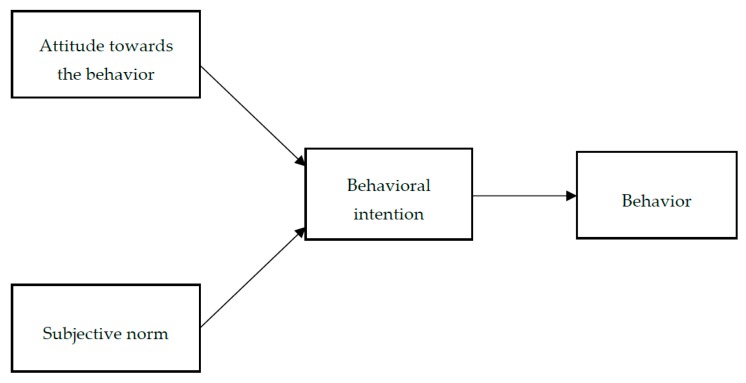
Theory of Reasoned Action (TRA). Source: Ajzen and Madden [23].

This study’s research model is anchored in the Theory of Reasoned Action (TRA), which is developed by Fishbein and Ajzen [21,22,23] (Figure 1). The TRA postulates that an individual’s behavioral intention, which is the immediate determinant of a certain behavior, is a function of two direct determinants of attitude (individual influence) and subjective norm (normative influence) [24]. Attitude denotes “the evaluation of an object, concept, or behavior along a dimension of favor or disfavor, good or bad, like or dislike” [25] (p. 3). Attitude has been important for research into consumer behavior and food consumption [26]. Subjective norm refers to “a person’s belief about whether significant others feel that he or she should perform the target behavior” [24] (p. 260). Subjective norms represent group influence or social pressure to perform the behavior [27,28,29]. The TRA has proved to be an effective model that can explain and predict consumer attitude, intention, and behavior relating to food products including functional foods [30,31,32,33].

**Figure 2:**
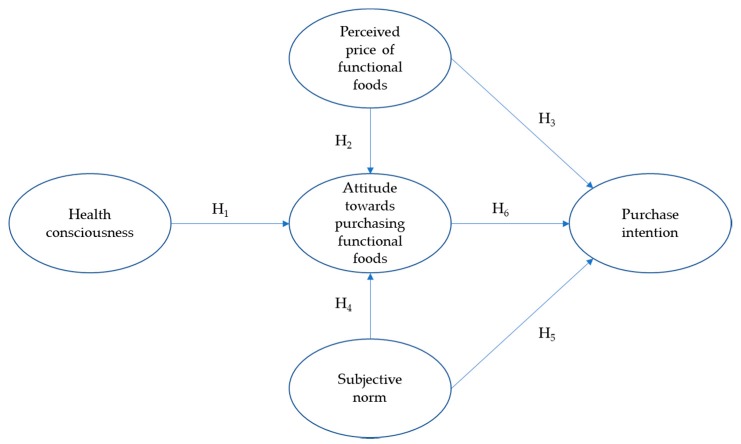
Research model.

Given that subject norm was found to be a weak determinant of intention, several authors have suggested to examine the linkage between subjective norm and attitude [19,34,35]. Our research model, therefore, sought to extend the TRA by positing that subjective norm has a positive influence on attitude. Such an extension will expectedly strengthen the model fit [34,36]. The model also incorporated health consciousness as a determinant of attitude towards purchasing functional foods. Such a construct appears to be an important driver of attitude about the purchase of healthy food products [16,37,38,39]. Moreover, price perception was included in our research model since the price of functional foods can play a negative role in the formation of attitude and purchase intention [16,39,40]. Understanding the barriers to functional foods purchase and acceptance (e.g., price) is essential to increasing consumer demand for these products [30]. Figure 2 depicts our research model. The hypothetical relationships between the constructs are explained and discussed in subsequent sections.

### 2.2. Development of Hypotheses

#### 2.2.1. Health Consciousness

Jayanti and Burns [41] refer to health consciousness as “the degree to which health concerns are integrated into a person’s daily activities” (p. 10). It also reflects an individual’s willingness to undertake health behaviors [42]. Consumers who are conscious about their health tend to engage in different health activities [43]. It is believed that functional foods promote health benefits in several essential parts of human physiology and decrease the risk of several diseases [5,44]. Many studies demonstrate that health consciousness and concern play an important role in consumer decision towards functional food products [45,46,47,48]. Chen [49] asserts that health conscious consumers who have modern health worries exhibit a more positive attitude about functional foods. Huang et al. [16] discover a positive relationship between health consciousness and attitude about purchasing functional foods. Hence, we have suggested the following hypothesis:

**Hypothesis** **1.** Health consciousness is positively related to attitude towards purchasing functional foods.

#### 2.2.2. Price

Price, representing monetary cost for consumers, is a key factor influencing consumer behavior associated with food products including functional foods [16,50]. Consumers generally perceive that functional foods have high prices and such a perception often deteriorates the acceptance and the sales of these products [1,6]. Szakály et al. [51] suggest that consumers’ price perception of functional foods might negatively affects their attitude about such products. Prior research indicates there is a (weak) negative association between price and purchase attitude associated with functional foods [16]. Given that price is considered as an important attribute of functional foods [52,53,54], it is argued that the perceived high price of these products is likely to exert a negative impact on consumer overall evaluation, i.e., attitude towards the purchase of functional foods.

According to Jaeger [50], price perception can influence food purchase in a way that the greater the price perceived by consumers, the lower their intentions to purchase foods. A conjoint study conducted by Ares et al. [55] concludes that increasing the price of functional yogurts might decrease consumer willingness to choose these products. In addition, regression analysis conducted by Huang et al. [16] reveals that perceived price has a significantly negative impact on intention to purchase functional foods. On the basis of the aforementioned discussion, we have proposed the below hypotheses:

**Hypothesis** **2.** Perceived price of functional foods is negatively related to attitude towards purchasing functional foods.

**Hypothesis** **3.** Perceived price of functional foods is negatively related to intention to purchase functional foods.

#### 2.2.3. Subjective Norm

Subjective norm represents “the perceived normative expectations of relevant reference groups or individuals” to perform certain behaviors [28] (p. 5). Several studies examining food consumption claim that subjective norm significantly enhances attitude [19,35]. A possible explanation is that reference groups and opinions of people who are important to customers can play an integral role in their evaluation of products and/or the purchase of such products [56]. A consumer survey reveals that subjective norm is positively correlated with attitude towards functional foods and vitamin supplements [57]. Sukboonyasatit [8] conducts a structural equation modeling and discover that subjective norm has a positive effect on attitude associated with consuming functional foods.

According to the TRA, subjective norm is the key motivator of behavioral intention. This is confirmed by O’Connor and White [57] who report that subjective norms encourage consumer willingness to trial functional foods. In addition, while Sukboonyasatit [8] asserts that subjective norm increases consumer intention to eat/drink functional foods, Rezai et al. [30] highlight that subjective norm has a direct impact on intention to buy natural functional foods. Based on the aforementioned discussion, we have proposed the following hypotheses:

**Hypothesis** **4.** Subjective norm is positively related to attitude towards purchasing functional foods.

**Hypothesis** **5.** Subjective norm is positively related to intention to purchase functional foods.

#### 2.2.4. Attitude

Behavioral attitude denotes the overall evaluation (either positive or negative) of the consequences relating to performing a certain behavior such as purchasing functional food products [9]. Attitude consists of both utilitarian (functional) and hedonic (emotional) facets [58]. Authors such as Rezai et al. [30], Sukboonyasatit [8], Nystrand and Olsen [9], and Patch et al. [59] conclude that consumers’ attitudes towards consuming functional foods lead to their intentions to consume or purchase such products (e.g., natural functional foods and those with added omega-3 oils). Recently, Huang et al. [16] find that purchase attitude had the strongest effect on intention towards buying functional foods. Hence, we have formulated the following hypothesis:

**Hypothesis** **6.** Attitude towards purchasing functional foods is positively related to purchase intention.

## 3. Research Methods

### 3.1. Focused Product Category

Factors affecting consumers’ attitudes and intentions towards food products often differ between different product types and categories [37,60]. In this study, we intentionally selected functional yogurts as the investigated product category. According to Euromonitor International [20], the sales of functional yogurt products doubled from 1322 billion VND in 2013 to 2663 billion VND in 2018. This dynamic product category has received growing interest from both domestic and international companies such as Vinamilk, Friesland Campina Vietnam, Nestle, Nutifood Nutrition Food, and Yakult. These companies have sought to expand their product lines to serve a wide range of market segments including consumers of different ages and incomes. Popular products include yogurts with specific nutrition for the development of children, probiotic yogurts for improving digestive system and health, fortified drinking yogurts with different flavors and yogurts with added vitamins, calcium and zinc. The market size of functional yogurts in Vietnam is expected to increase progressively during the next decade owing to sustainable investment in production and marketing efforts from the key companies [20].

### 3.2. Measures and Sample

Items used to operationalize the constructs in our research model were selected and adapted from prior literature. The measures of health consciousness and price perception were adapted from measures suggested by Huang et al. [16] and Nguyen et al. [37]. Subjective norms and intention to purchase functional yogurts were measured using items contained in Sukboonyasatit [8]. Additionally, three items operationalizing attitude were adapted from Huang et al. [16] and Voss et al. [58]. Respondents rated all the measurement items based on a 7-point Likert scale, ranging from 1 for ‘strongly disagree’ to 7 for ‘strongly agree’. Following the back-translation technique recommended in previous research [29,61,62], the items, originally in English, were translated into the target language of Vietnamese. Specifically, a team consisting of two professional translators and two bilingual marketing lecturers was recruited to conduct the back translation, cross-check and confirm the translated versions. Twenty consumers of functional foods were invited to participate in a pilot test to confirm the clarity and meanings of measurement items. The final questionnaire used in the present study has 4 parts: (1) Introduction presenting an overview of the research, (2) Screening questions for recruiting eligible respondents, (3) Statements measuring the variables in the research model, and (4) Demographic questions. In part (2), while one screening question was used to recruit respondents who had involved in buying functional foods, the other concerned their interest in functional yogurts.

Data were collected from Vietnamese consumers, aged 18 and over, who were interested in functional yogurts using interviewer-administered questionnaire. Survey method has been prevalent in marketing and consumer research as it enables the investigation of certain characteristics of a population by obtaining data from a sizable sample of the population [63,64]. The questionnaire was administered in several supermarkets and grocery stores in Hanoi, which are the major distributors of functional foods [20]. Hanoi, situated in the North of Vietnam, is the capital city which has the second-highest population density with 2398 persons per km^2^ [17]. Most of major supermarkets and traditional grocery retailers are located in Hanoi. In addition, this city has been chosen as the research site in prior studies on food consumption because consumers in Hanoi generally have higher income levels and a healthier lifestyle [18,37]. The data collection followed the protocol that was reviewed and approved by the Department of Research Administration at Thuongmai University. Six research assistants supervised by the researcher approached consumers who shopped at the stores and request their voluntary participation. The research assistants informed consumers who agreed to provide responses about the research objectives and how their privacy and anonymity would be ensured. During the period from March to May 2019, 604 questionnaires were completed. The data were checked for outliers and normality of distribution. The Mahalanobis distance indicated that 8 questionnaires contained multivariate outliers. These questionnaires were removed, resulting in a final effective sample of 596 cases. The demographic profiles of the respondents are depicted in Table 1.

According to the 2019 Census [17], the population sex ratio of Vietnam is 99.1 males per 100 females countrywide and the ratio is 96.5 males per 100 females in urban areas, which is similar to that reported in our sample. In addition, the marriage incidence of people aged 15 and over is 69%, which is slightly higher than that obtained in our sample. However, it should be mentioned that our study focused on respondents aged 18 and above. Furthermore, the majority of our respondents were young adults and highly educated consumers, which closely represents the population. Hence, it is reasonable to conclude that our sample is fairly representative of the national profile data with respect to gender, age, education and marital status.

### 3.3. Analysis Method

We used SPSS Statistics and AMOS 24 (IBM, Armonk, NY, USA) to analyze the data and examine the hypothetical associations between the constructs in the proposed model. SPSS was applied to produce means, standard deviations, bivariate correlations and reliability statistics. We used AMOS to conduct confirmatory factor analysis (CFA) and then perform structural equation modelling (SEM). We selected SEM because it advances ordinary regression models by enabling the assessment of complex models containing different direct and indirect relationships [65,66]. The CFA results enable us to calculate composite reliability (CR), average variance extracted (AVE) and square root of AVE to assess the validity of the measures. Subsequently, SEM was conducted to assess the model fit and test the proposed. The goodness-of-fit measures used to assess the model fit included normed chi-square (χ^2^/df), goodness-of-fit index (GFI), comparative fit index (CFI), normed fit index (NFI), Tucker–Lewis index (TLI), and root mean square error of approximation (RMSEA).

## 4. Analysis and Results

### 4.1. Descriptive Statistics, Reliability, and Validity

Table 2 demonstrates the items’ descriptive statistics and the constructs’ reliability that is assessed through Cronbach alpha coefficient values (α). As shown in Table 2, all the alpha values were greater than the generally agreed threshold of 0.7 [67], indicating the consistency between the items measuring each construct.

Results of the CFA indicated that the measurement model had good fit with χ^2^/df = 1.298, GFI = 0.977, CFI = 0.992, NFI = 0.966, TLI = 0.989, and RMSEA = 0.022 since these values met the thresholds recommended by Kline [68] and Hair et al. [67]. Based on the CFA results, CR and AVE were calculated and illustrated in Table 3.

As shown in Table 3, CR was greater than 0.7 and AVE was higher than 0.5. In addition, standardized factor loadings, ranging from 0.687 to 0.812 (Table 2), were above the suggested value of 0.6. The convergent validity of the constructs and measures was therefore ensured [67,68]. Table 3 also indicates that the square root of the AVE for each variable (0.735–0.764) was greater than its correlations with other variables examined in the research model. This confirmed the discriminant validity of the constructs as suggested by Byrne [69] and Fornell and Larcker [70]. Moreover, all correlation coefficients between the constructs were below 0.6, eliminating possible problems of multi-collinearity [71].

### 4.2. Structural Model Analysis and Hypotheses Testing

The goodness-of-fit indices of the structural model is shown in Table 4. It can be concluded that the structural model demonstrated a good fit to the sample data, with χ^2^/df = 1.386, GFI = 0.975, CFI = 0.989, NFI = 0.963, TLI = 0.986, and RMSEA = 0.025. The model explained a significant 35% (*R*^2^ = 0.35) of the variance in consumers’ attitudes towards functional yogurts and 24% (*R*^2^ = 0.24) of the variation in their intention to consume functional yogurts

The path analysis is summarized in Table 5. We found that almost all the relationships between the investigated constructs were significant (albeit at different significant levels), except that between price and purchase intention. Health consciousness significantly affected attitude towards purchasing functional yogurts (*β* = 0.234, *p* < 0.001). As expected, the relationship between price and attitude was negative and significant (*β* = −0.112, *p* < 0.05). However, the negative impact of price perception on purchase intention was insignificant (*β* = −0.005, *p* > 0.05). Furthermore, subjective norms significantly influenced attitude (*β* = 0.468, *p* < 0.001) and intention towards purchasing functional yogurts (*β* = 0.201, *p* < 0.01). Finally, there was a significant and positive association between attitude and purchase intention (*β* = 0.353, *p* < 0.001).

## 5. Discussion and Implications

Our study has identified several key factors and investigated their impact on consumer attitude and intention relating to the purchase of a specific type of functional foods (i.e., functional yogurts) in the emerging market context of Vietnam. The SEM results demonstrate that health consciousness exerts a significant and positive effect on attitude towards purchasing functional yogurts. This finding is similar to that of prior research highlighting the integral role of health consciousness or concerns in motivating consumer perception and attitude associated with the purchase and consumption of healthy and functional foods in developed countries (e.g., Sweden and Germany) [72,73] and emerging countries (e.g., China, Malaysia, and Taiwan) [16,74,75]. Rising health consciousness has also been identified as the major driver of the development of functional food products in Vietnam [20].

Another key finding is that consumer perceived price of functional yogurts reduces attitude towards purchasing these products. In general, consumers take price into consideration while evaluating functional foods and the purchase of such products [54,76]. The perceived high price of functional yogurts therefore can contribute to negative evaluation of functional yogurts, lowering their purchase attitude. Strikingly, price does not significantly affect consumer intention to buy functional yogurts. This finding contradicts with that of Huang et al. [16] who find that price is the direct inhibitor of consumer intention to purchase functional foods in China. It also challenges the widespread belief that consumers in developing countries like Vietnam generally have financial constraints [77,78], which expectedly reduce their willingness to pay more for environmentally friendly and healthy products such as functional foods. A possible explanation for our interesting finding relates to the focused product category. According to Euromonitor International [20], while Vietnamese consumers believe that functional yogurts offer great health benefits, the prices of such products are not remarkably higher than those of conventional yogurts. Several prior studies emphasize that consumers are willing to give up on price for proven health benefits of functional foods [14,79,80], leading to their willingness to purchase and consume these products. Manufacturers and retailers that take aim at the functional yogurt market in Vietnam should particularly leverage on this finding to market their products more effectively.

The results also show that subjective norm is the key motivator of both attitude and purchase intention associated with functional yogurts. This finding is in line with the study conducted by Sukboonyasatit [8] who examined consumer intention to use functional foods in New Zealand. It also extends the TRA and previous functional food studies done in Norway and Malaysia that only confirm the positive relationship between subjective norm and behavioral intention [9,30,75]. It should be also noted that among the determinants of attitude, subjective norm has the greatest influence. This can be explained by the fact that Vietnam is a highly collectivist culture where consumers value interpersonal relationships and their behaviors are strongly motivated by subjective norm and social pressure [81,82]. The final major finding is the positive association between attitude and purchase intention. Importantly, attitude appears to be the most effective predictor of consumer intention to purchase functional yogurts. This finding supports the TRA and echoes several relevant studies [8,9,16]. The positive impact of both subjective norm and attitude on purchase intention can also partly explain the insignificant relationship between price and intention. That is, despite the relatively high price of functional foods, consumers generally are willing to purchase these products because of stronger influences from social groups and their overall attitudes.

The findings of our study have several important practical implications. Education and communication programs that aim at increasing consumers’ health consciousness and their awareness of the health benefits of functional foods are key to the success of products such as functional yogurts. Food manufacturers, retailers, policymakers, and other relevant organizations must provide target and potential consumers with clear and honest information about how functional foods such as probiotic yogurts influence people’s biological response in their body, improve their state of well-being, and/or reduce specific diseases. In addition, marketing communication programs need to highlight how the purchase and consumption of functional yogurts can contribute to and demonstrate consumers’ healthy lifestyles. Such information and messages can be communicated to consumers through mass media, social media, educational programs, food package, and in-store channels (e.g., brochures, leaflets, and salespersons). With regard to social media platforms, YouTube and Facebook should be utilized as they are the leading social media in Vietnam [83].

Given the important role of subjective norms in enhancing consumer purchase attitude and intention, marketing programs should feature social influencers or functional food experts (e.g., doctor, health consultants, and food experts). Moreover, advertisements can portray family and other influential groups that expect consumers to purchase functional yogurts. Word-of-mouth can also be utilized in the forms of functional food forums or referrals. It is also important to reduce the product price, lowering consumer monetary costs associated with the purchase of functional yogurts. In this respect, manufacturers need to improve their production efficiency while retailers should make every effort to reduce their distribution costs. Finally, other programs such as bundle pricing (e.g., family pack of yogurts) or discounts should be also utilized to encourage consumer to purchase more functional foods including functional yogurts.

## 6. Conclusions and Future Research

This study is among the first attempt to examine consumer attitude and intention with respect to purchasing functional yogurts in Vietnam, which is an important Southeast Asian emerging economy and a promising market for healthy and functional foods. Our validated model linking health consciousness and price perception to the elements of TRA is unique, and it can serve as a theoretical background for future studies. The findings reveal that while health consciousness and subjective norm enhance purchase attitude, perceived price has a negative impact on such attitude. Moreover, subjective norm and attitude positively affect intention to purchase functional yogurts. Importantly, subjective norm is the strongest predictor of attitude which in turn has the greatest effect on purchase intention. These findings enrich the extant literature and improve the understanding of how different factors affect consumer attitude and intention towards purchasing functional yogurts.

Our study, however, has several limitations. First, other relevant factors such as trust, self-efficacy, and environmental concern were not included in the research model. Future research could extend our model by including these exogenous variables. Second, our study is confined to functional yogurts in Vietnam. Future research could therefore test and validate our model in other contexts, such as vitamin supplements, functional cereals, or biscuits in other emerging countries (e.g., China, India, Indonesia, and Malaysia). Due to the lack of an effective sampling frame, our study utilized non-probability sampling. Future research could address this by using probability sampling techniques. Finally, future research could conduct a longitudinal study to examine consumer actual purchase of functional foods and compare the differences between different social and demographic segments (e.g., age, gender, education, income levels, and family types) with respect to their intentions, purchases, and consumption towards functional foods.

## Figures and Tables

**Table 1:** Sample profile.

Gender	N	%
Female	313	52.5
Male	283	47.5
Age		
18–29	148	24.8
30–39	175	29.4
40–49	137	23.0
50 and above	136	22.8
Marital Status		
Single/never married	172	28.3
Currently married	376	61.7
Widowed	34	5.6
Divorced/separated	27	4.4
Education		
High school or lesser	74	12.4
Vocational school	93	15.6
College	118	19.8
University undergraduate	225	37.8
Postgraduate	86	14.4
Employment		
Full-time	346	58.0
Part-time	130	21.8
Retired	66	11.1
Unemployed	54	9.1
Household Monthly Income		
Under VND 5,000,000	56	9.4
VND 5,000,000–10,000,000	121	20.3
VND 10,000,001–20,000,000	231	38.8
VND 20,000,001–30,000,000	82	13.7
VND 30,000,001–40,000,000	46	7.7
Over VND 40,000,000	60	10.1
Total	596	100

**Table 2:** Constructs, items, and properties.

Variables and Items	Mean	SD	FLs	α
*Health consciousness (adapted from [16,37])*				0.794
I am prepared to do anything that is good to health	4.83	1.431	0.780	
I often dwell on my health	4.93	1.385	0.702	
I think that I take health into account a lot in my life	4.83	1.464	0.769	
*Perceived price (adapted from [16,37])*				0.807
Functional yogurts are expensive	4.21	1.478	0.743	
The price of functional yogurts is a barrier to consume them	4.17	1.485	0.794	
People should purchase functional yogurts, even though they are more expensive than conventional yogurts	4.13	1.457	0.753	
*Subjective norms (adapted from [8,16])*				0.780
My family thinks I should purchase functional yogurts	4.93	1.462	0.764	
My friends or colleagues think that I should purchase functional yogurts	4.92	1.463	0.703	
My doctor or health expert thinks that I should purchase functional yogurts	4.84	1.402	0.744	
*Attitude (adapted from [16,58])*				0.798
Purchasing functional yogurts regularly would be …				
Bad-Good	4.97	1.301	0.742	
Unpleasant-Pleasant	4.92	1.310	0.769	
Foolish-Wise	4.92	1.377	0.753	
*Intention (adapted from [8,16])*				0.776
I intend to purchase functional yogurts in the next few weeks	4.91	1.259	0.687	
I aim to purchase functional yogurts for my healthy lifestyle	4.98	1.259	0.701	
I plan to purchase functional yogurts	5.08	1.290	0.812	

Note: each item’s value ranged from 1 to 7.

**Table 3:** Correlations, Composite Reliability (CR), and Average Variance Extracted (AVE).

Constructs	CR	AVE	(1)	(2)	(3)	(4)	(5)
(1) Health consciousness	0.795	0.564	0.751				
(2) Price	0.807	0.583	−0.098	0.764			
(3) Subjective norms	0.781	0.544	0.224	−0.071	0.737		
(4) Attitude	0.799	0.570	0.342	−0.168	0.530	0.755	
(5) Intention	0.778	0.541	0.294	−0.068	0.386	0.450	0.735

**Table 4:** Goodness-of-fit indices.

Models	χ^2^/df	GFI	CFI	NFI	TLI	RMSEA
Thresholds	<3	>0.9	>0.9	>0.9	>0.9	<0.05
Measurement model	1.298	0.977	0.992	0.966	0.989	0.022
Structural model	1.386	0.975	0.989	0.963	0.986	0.025

**Table 5:** Path Analysis and Hypotheses.

Hypotheses	β	*t*-Value	*p*-Value	Findings
H_1_: Health consciousness → Attitude	0.234	4.777	***	Accepted
H_2_: Price → Attitude	−0.112	−2.443	*	Accepted
H_3_: Price → Intention	−0.005	−0.109	0.913	Rejected
H_4_: Subjective norms → Attitude	0.468	8.506	***	Accepted
H_5_: Subjective norms → Intention	0.201	3.232	**	Accepted
H_6_: Attitude → Intention	0.353	5.434	***	Accepted

Note: *** *p* < 0.001; ** *p* < 0.01; * *p* < 0.05.
